# Anti-CTLA4 monoclonal antibodies: the past and the future in clinical application

**DOI:** 10.1186/1479-5876-9-196

**Published:** 2011-11-13

**Authors:** Paolo A Ascierto, Francesco M Marincola, Antoni Ribas

**Affiliations:** 1 Unit of Medical Oncology and Innovative Therapies, Istituto Nazionale Tumori Fondazione Pascale, Naples, Italy; 2 Infectious Diseases and Immunogenetics Section (IDIS), Department of Transfusion Medicine, Clinical Center and Center for Human Immunology (CHI), NIH, Bethesda, Maryland, USA; 3 Department of Medicine, Jonsson Comprehensive Cancer Center at the University of California Los Angeles (UCLA), Los Angeles, California, USA

## Abstract

Recently, two studies using ipilimumab, an anti-CTLA-4 monoclonal antibody (mab) demonstrated improvements in overall survival in the treatment of advanced melanoma. These studies utilized two different schedules of treatment in different patient categories (first and second line of treatment). However, the results were quite similar despite of different dosage used and the combination with dacarbazine in the first line treatment. We reviewed the result of randomized phase II-III clinical studies testing anti-CTLA-4 antibodies (ipilimumab and tremelimumab) for the treatment of melanoma to focus on practical or scientific questions related to the broad utilization of these products in the clinics. These analyses raised some considerations about the future of these compounds, their potential application, dosage, the importance of the schedule (induction/manteinance compared to induction alone) and their role as adjuvants. Anti-CTLA-4 antibody therapy represents the start of a new era in the treatment of advanced melanoma but we are on the steep slope of the learning curve toward the optimization of their utilization either a single agents or in combination.

## Introduction

CTLA-4-blocking antibodies are fully human novel monoclonal antibodies directed against CTLA-4. By targeting CTLA-4 these antibodies prevent the interaction between the costimulatory molecules B7.1 an B7.2 (CD80 and CD86) and linking to CTLA-4, thus removing the CTLA4 inhibitory signal and releasing a brake on the immune system. This allows a natural immune response to react to cancer cells. The mechanism of action of anti-CTLA-4 antibodies is therefore indirect, through enhancing T-cell mediated immune responses.

Two anti-CTLA-4 antibodies have been tested in advanced clinical trials, either in phase II and phase III: ipilimumab and tremelimumab. There is a very small difference between the two products: both are fully human monoclonal antibodies directed against CTLA-4, but ipilimumab is an immunoglobulin IgG1 isotype and tremelimumab is a non-complement-fixing IgG2 isotype.

## Ipilimumab phase II studies: the assessment of the treatment schedule

Ipilimumab has being extensively studied in different phase II trials. In a phase II randomized study, patients with metastatic melanoma received different doses of ipilimumab (0.3 vs 3 vs 10 mg/kg) and the results indicated a statistically significant trend of increased response rates with increased dose, suggesting a dose-effect [1]. Overall, most promising results in terms of best overall response rate (BORR) were obtained with 10 mg/kg of ipilimumab, every 3 weeks for a total of 4 doses (induction phase) followed by maintenance period in which ipilimumab was administrated every 12 weeks (maintenance phase). This was the reason for the choice of such a schedule for the front line phase 3 study. The most common treatment-related adverse events (AEs) associated with the use of ipilimumab were immune-related and specific algorithms have been subsequently developed, showing that early recognition and correct therapeutic approach with steroid therapy make most of these AEs manageable and reversible [2].

## Ipilimumab phase III studies

In 2010, results from the MDX010-20 clinical trial were published [3]. This is the first randomized phase III trial to have demonstrated a benefit in overall survival (OS) in pretreated patients with metastatic melanoma. This study showed the superiority of ipilimumab arm compared to a gp100 vaccine arm: ipilimumab monotherapy had a median OS survival of 10.1 months whereas the OS for gp100 monotherapy was only 6.4 months. This clinical trial was activated in 2004, before the data from the dose-ranging phase II randomized trial were available. It used an induction regimen of 3 mg/kg of ipilimumab once every 3 weeks for four administrations; patients showing disease progression after either a stable disease lasting more than 3 months after week 12 or a confirmed partial or complete response were eligible for additional courses of therapy. The safety profile in this study was consistent with the prior studies with ipilimumab.

On June 2011, results of a second phase III trial comparing dacarbazine versus dacarbazine plus ipilimumab (CA184-024 study) in treatment naïve patients with metastatic melanoma were published [4]. Ipilimumab was administered at a dose of 10 mg/kg every 3 weeks for 4 doses, followed by maintenance therapy with 10 mg/kg ipilimumab for eligible patients. This study, although less than expected, supported the results of the previous phase III trial by showing an OS of 11.2 months for patients treated with dacarbazine plus ipilimumab and an OS of 9.1 months for patients treated with dacarbazine alone.

## Ipilimumab efficacy: Optimal dose and schedule

**Figure 1:**
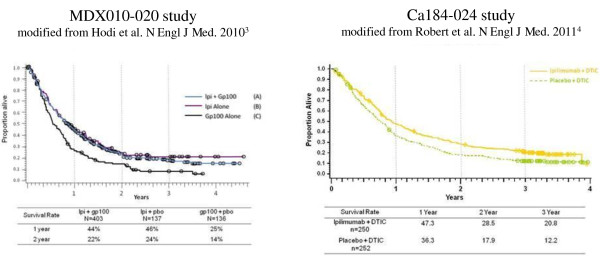
**Comparison between the Overall Survival curves of the studies MDX010-20 and CA184-024**.

Even if they are not directly comparable, given the differences in study design, by looking at the survival curves of the two phase III trials [3,4] they appear to be quite similar (Figure 1), although there is less evidence of a tail of the curve of long term durable responses in the trial combining ipilimumab with dacarbazine. This raises some questions about which are the best dosage (3 vs 10 mg/kg), the best schedule (re-induction vs maintenance) and the combination with chemotherapy. Another question concerns the role of dacarbazine regarding combination regimes and toxicity profile, since in the dacarbazine plus ipilimumab arm hepato-toxicity appeared to be higher than in the control arm (31,6% vs 2,4% of grade 3/4) and higher than with the prior experience with single agent ipilimumab at 3 or 10 mg/kg. In this regard, results from another combination trial with fotemustine could give us more information about efficacy and safety of combination regimens with cytotoxic chemotherapy [5].

### Optimal dose

Regarding the best dose between 3 and 10 mg/kg, on the base of effect-response analysis in phase II and phase III studies, it is not fully elucidated if the dose of 3 mg/kg ipilimumab is the optimal one. Certainly, taking into account data coming from the expanded access program (EAP), 3 mg/kg seems to be sufficient to be clinically effective and does not seem to be burdened by excessive toxicity [6]. Nevertheless, in the CA184-024 study the used ipilimumab dose was 10 mg/kg and, in the dacarbazine plus ipilimumab arm, only 93 patients (36,8%) completed all four induction cycles. Therefore, no conclusion about which is the best regimen can be drawn at the present moment. An upcoming randomized trial will directly compare 3 mg/kg versus 10 mg/kg ipilimumab in patients with advanced melanoma and will probably help us to answer the question about efficacy in terms of OS and safety of the two regimens.

It is also clear that the two schedules have different safety profiles. The incidence of severe (grade 3-4) adverse events (AEs) in the 3 mg/kg schedule was 10-15%, while in the ipilimumab combination arm of the CA184-024 study was 56.3% (41.7% was severe immuno-related AEs, irAEs). It is also clear that in the future we will need to monitor the incidence of severe irAE even considering new possible toxicities (neutropenia, thrombocytopenia) [7,8] because the early recognition and treatment is mandatory to reduce the risk of sequelae for patients.

### Maintenance or re-induction?

One of the main points to be clarified is the optimal treatment schedule and the role of re-induction or maintenance. In fact, in the first line clinical trial only 43 patients (17.2%) received at least one dose of maintenance treatment, while in the second line trial just 7% of patients received at least one re-induction dose. Moreover, if the true value of maintenance remains to be defined, the utility of reinduction has been studied. In the MDX010-20 study [3], 21 out of 31 (68%) patients who received reinduction with ipilimumab had a response or a durable SD with reinduction without significant additional toxicities. Therefore, it remains to be defined whether 4 cycles are sufficient in terms of efficacy and whether re-induction or maintenance do add anything in terms of efficacy or could alternatively worsen toxicities.

Additional evidence regarding the utility of reinduction is derived from the MDX010-08 trial which compared ipilimumab alone (3 mg/kg for 4 cycles schedule) versus ipilimumab in combination with dacarbazine [9]. In this trial, patients who progressed on monotherapy could cross-over and receive combination therapy upon progression, but none of the patients treated with the cross-over had a response. Moreover, in this trial the BORR and the disease control rate (DCR) (see table 1) was quite similar to the phase III studies: in the ipilimumab alone arm, the BORR was 5.4% and the DCR was 21.6% (the BORR and DCR of the ipilimumab plus gp100 arm in MDX010-20 study was respectively 5.7% and 20.1%) [3]; while, in the dacarbazine plus ipilimumab combination arm, the BORR was 14.3% and the DCR was 37.1% (the BORR and DCR of the dacarbazine plus ipilimumab arm in the CA184-024 study was respectively 15.2% and 33.2%) [4].

**Table 1:** Comparison among the Best Overall Response Rate, the Disease Control Rate, and the duration of response of the three randomized phase II-III studies which utilized ipilimumab (MDX010-08, MDX010-20, and CA184-024)

	MDX010-08		MDX010-20			CA184-024	
	DTIC + Ipi	Ipi	Ipi + gp100	Ipi	gp100	DTIC + Ipi	DTIC
**Patients (N.)**	(35)	(37)	(403)	(137)	(136)	(250)	(252)
							
**BORR**	14.3%	5.4%	5.7%	10.9%	1.5%	15.2%	10.3%
							
**DCR**	37.1%	21.6%	20.1%	28.5%	11.0%	33.2%	30.1%
**Median Duration of BOR in months (CI)**	5.7* (0.7-19.2)	10.8* (3.0-25.4)	11.5 (5.5-NR)	NR (28.1-NR)	NR (2.0-NR)	19.3 (not reported)	8.1 (not reported)

*this value indicated the major durable DCR (≥ 24 weeks)

**DTIC **= dacarbazine

**Ipi **= ipilimumab

**BORR **= Best Overall Response Rate = Complete Remission + Partial Remission

**DCR **= Disease Control Rate = Complete Remission + Partial Remission + Stable Disease

**BOR **= Best Overall Response

**CI **= Confidence Interval

**NR **= Not Reached

## Evaluation of response

Another important issue concerns how to evaluate the response to anti-CTLA-4 therapy. In fact, if prolongation of survival is a clear fact, it still remains unsolved the problems of response evaluation. In fact, the main difficulty for several oncologists without experience in immunotherapy will be the evaluation of response in patients treated with ipilimumab. As described, ipilimumab doesn't impact on PFS and BORR. For this reason could be difficult to take a decision, for an oncologist, at the time of the first assessment (normally oncologist retain that the week 12 represent the best time for this). Immuno-related response criteria (irRC) could help oncologists in evaluating the response to ipilimumab. However, it remains not easy to perform an assessment when it's not clearly evident a response and considering the limited percentage of complete and partial response.

For this reason, it merits a comment the study design of the EORTC18071, an ongoing study on ipilimumab in the adjuvant setting. The primary endpoint of such trial, is relapse-free survival (RFS) and the used dose is 10 mg/kg with induction and 3-years maintenance. This study underlines the above consideration that, if the impact of anti-CTLA-4 therapy is clearly visible in terms of OS, so it cannot be said if we consider the surrogates (BORR and DFS). Moreover, while in the MDX010-20 trial both BORR and PFS are statistically significant in the ipilimumab arms, in the CA184-024 trial progression free survival (PFS) is similar in the two arms. Is it therefore RFS the best way to show efficacy of ipilimumab in the adiuvant setting? Probably not, but at the moment it is not easy to find a valid surrogate for OS.

The duration of response is another important parameter. In table 1 we summarized the results in the three randomized phase II-III trial. It's clear enough that ipilimumab gives durable response.

## The Tremelimumab experience

With regard to the other anti-CTLA-4 antibody tremelimumab, treatment schedule of 15 mg/kg every 90 days was defined in different phase I/II studies [10,11] and therefore tested in a phase III trial versus chemotherapy control arm (dacarbazine or temozolomide) [12]. The 15 mg/kg dosing regimen every 90 days was chosen based on the results of a prior phase 2 randomized clinical trial comparing this dosing regimen with tremelimumab at 10 mg/kg administered monthly; results of response rate, PFS and OS was similar in both arms, while the safety profile favored the 15 mg/kg every 90 days dosing regimen [10]. Unfortunately, differences in OS in the phase 3 trial were not statistically significant with 11.76 months in the tremelimumab arm versus 10.71 months in the control arm. One of the reason could be the treatment schedule every 90 days, However, this is unlikely since comparison of the response rates (assessed by independent radiology review committees) between the two phase 2 single arm studies of ipilimumab at 10 mg/kg every 3 weeks and tremelimumab at 15 mg/kg every 90 days demonstrate (with the caveats of cross-study comparisons) very similar response and toxicity results [11,13]. Another possibility is that the restriction of the LDH level to up to twice the upper limit of normality in the tremelimumab phase 3 trial resulted in an advantage for the control arm that was not present in the ipilimumab phase 3 trials that did not have this restriction. More likely, the concurrent availability of ipilimumab in several studies that did not exclude prior anti-CTLA4-based therapies (including two expanded access protocols) allowed a significant cross-over to ipilimumab in patients randomized to the control arm of the tremelimumab phase 3 trial. Since the ipilimumab studies were blinded and all the tremelimumab studies excluded patients who had been previously enrolled in a study including an anti-CTLA4 antibody, cross-over to tremelimumab in the ipilimumab control arms was less likely.

## Identification of responder patients

An important challenge for the future will be the identification of patients who are more likely to respond to anti-CTLA4 treatment. It has been suggested that the absolute lymphocyte count (ALC) ≥ 1000/μL after 2 ipilimumab treatments (week 7) could be a possible marker and seems to correlate with clinical benefit and OS [14]. Another important biomarker could be the expression of the inducible costimulator (ICOS) molecule [15-17], a member of the immunoglobulin gene family, on T cells. ICOS was shown to correlate with clinical outcome in a small cohort of melanoma patients treated with ipilimumab [18] and to be necessary for optimal anti-tumor responses mediated by anti-CTLA-4 [19].

Other potential biomarkers could be the mean rate of CD8 change, the resistance of CD4+ T cells to Treg mediated inhibition in vitro, and the frequency of IL-17 secreting CD4+ T cells [20]. Even biomarkers in the tumor microenvironment have been demonstrated to be associated with clinical activity in patients treated with ipilimumab [21]. In fact, it was showed that clinical activity was related with high expression of FOXP3 and IDO at baseline and an increase from baseline in tumor-infiltrating lymphocytes (TILs) (Wk 4) in tumor biopsies. However, further studies should be designed to evaluate these biological endpoints and provide prospective validation.

## Conclusion

For more than 30 years we have been waiting for advances in the therapy of patients with advanced melanoma, while just over the last 2 years we have witnessed a real revolution. If ipilimumab can rightly be considered a cornerstone of a new era in cancer treatment, there is still a lot to be done to optimize the therapy with anti-CTLA-4 antibodies, especially to define the best schedule for next combination regimens (immunomodulatory antibodies, BRAF/MEK inhibitors, vaccines, etc.) which represent the natural evolution of future melanoma therapy [22].

## Competing interests

PAA participated to Advisory Board from Bristol Myers Squibb, Merck Sharp & Dohme, Roche-Genentech, GSK and received honoraria from Bristol Myers Squibb, Roche Genentech and Merck Sharp & Dohme; FM has no conflict of interest; AR received honoraria from Bristol Myers Squibb and was Consultant for Amgen, Merck, Roche-Genentech.

There are no financial disclosure and acknowledgements

## Authors' contributions

All Authors: 1) made intellectual contributions and participated in the acquisition, analysis and interpretation of data; 2) have been involved in drafting the manuscript; and 3) have given final approval of the version to be published.
